# Three-dimensional ultrasonography and power Doppler for discrimination between benign and malignant endometrium in premenopausal women with abnormal uterine bleeding

**DOI:** 10.1186/s12905-016-0297-3

**Published:** 2016-03-16

**Authors:** Mohamed El-Sharkawy, Akmal El-Mazny, Wafaa Ramadan, Dina Hatem, Aly Abdel-Hafiz, Mohamed Hammam, Adel Nada

**Affiliations:** Department of Obstetrics and Gynecology, Faculty of Medicine, Cairo University, Cairo, Egypt

**Keywords:** 3D ultrasonography, Doppler, Endometrial carcinoma, Hysteroscopy, Premenopausal bleeding

## Abstract

**Background:**

Ultrasonography has been extensively used in women suspected of having a gynecological malignancy. The aim of this study is to evaluate the efficacy of 3D ultrasonography and power Doppler for discrimination between benign and malignant endometrium in premenopausal women with abnormal uterine bleeding.

**Methods:**

This cross-sectional study included 78 premenopausal women with abnormal uterine bleeding scheduled for hysteroscopy and endometrial curettage. The endometrial thickness (ET), uterine artery pulsatility index (PI) and resistance index (RI), and endometrial volume (EV) and 3D power Doppler vascularization index (VI), flow index (FI), and vascularization flow index (VFI) were measured and compared with hysteroscopic and histopathologic findings.

**Results:**

The ET (*P* <0.001), EV (*P* <0.001), and endometrial VI (*P* <0.001) and VFI (*P* = 0.043) were significantly increased in patients with atypical endometrial hyperplasia and endometrial carcinoma (*n* = 10) than those with benign endometrium (*n* = 68); whereas, the uterine artery PI and RI and endometrial FI were not significantly different between the two groups. The best marker for discrimination between benign and malignant endometrium was the VI with an area under the ROC curve of 0.88 at a cutoff value of 0.81 %.

**Conclusion:**

3D ultrasonography and power Doppler, especially endometrial VI, may be useful for discrimination between benign and malignant endometrium in premenopausal women with abnormal uterine bleeding.

## Background

Endometrial carcinoma is the most common form of gynecologic cancer in developed countries, and it is the fourth most common malignant tumor among women worldwide [1]. Abnormal uterine bleeding is usually the first symptom; therefore, appropriate evaluation of women with premenopausal or postmenopausal bleeding will allow for early diagnosis of endometrial carcinoma and the best opportunity for cure [2].

Ultrasonography has been extensively used in women suspected of having a gynecological malignancy, especially in ovarian [3] and endometrial [4] cancer. In fact, transvaginal ultrasonography is considered the initial imaging procedure for evaluating abnormal vaginal bleeding due to its ability to depict endometrial pathology, its widespread availability, and its excellent safety profile and cost effectiveness [5].

Three-dimensional ultrasonography is a new imaging technique that has become currently available in gynecologic practice [6], specifically in gynecologic oncology [7]. In addition, 3D power-Doppler ultrasonography allows a 3D reconstruction of the vascular network and also calculating vascular indices based on the total and relative amount of power Doppler information within the volume of interest [8].

The aim of this study is to evaluate the efficacy of 3D ultrasonography and power Doppler for discrimination between benign and malignant endometrium in premenopausal women with abnormal uterine bleeding.

## Methods

This cross-sectional study was conducted at the Department of Obstetrics and Gynecology, Faculty of Medicine, Cairo University, during the period from August 2013 to May 2014. The study protocol was approved by the Research Ethics Committee, and informed verbal consent was obtained from all participants.

The study population consisted of 78 premenopausal women with abnormal uterine bleeding scheduled for hysteroscopy and endometrial curettage. They were subjected to detailed history taking, complete general and gynecological examination, routine pre-operative laboratory investigations, and preliminary transvaginal ultrasound. The exclusion criteria included uterine fibroids, adenomyosis, endometrial polyps, and any general diseases, hormones or medications that could potentially affect pelvic blood flow.

**Fig. 1:**
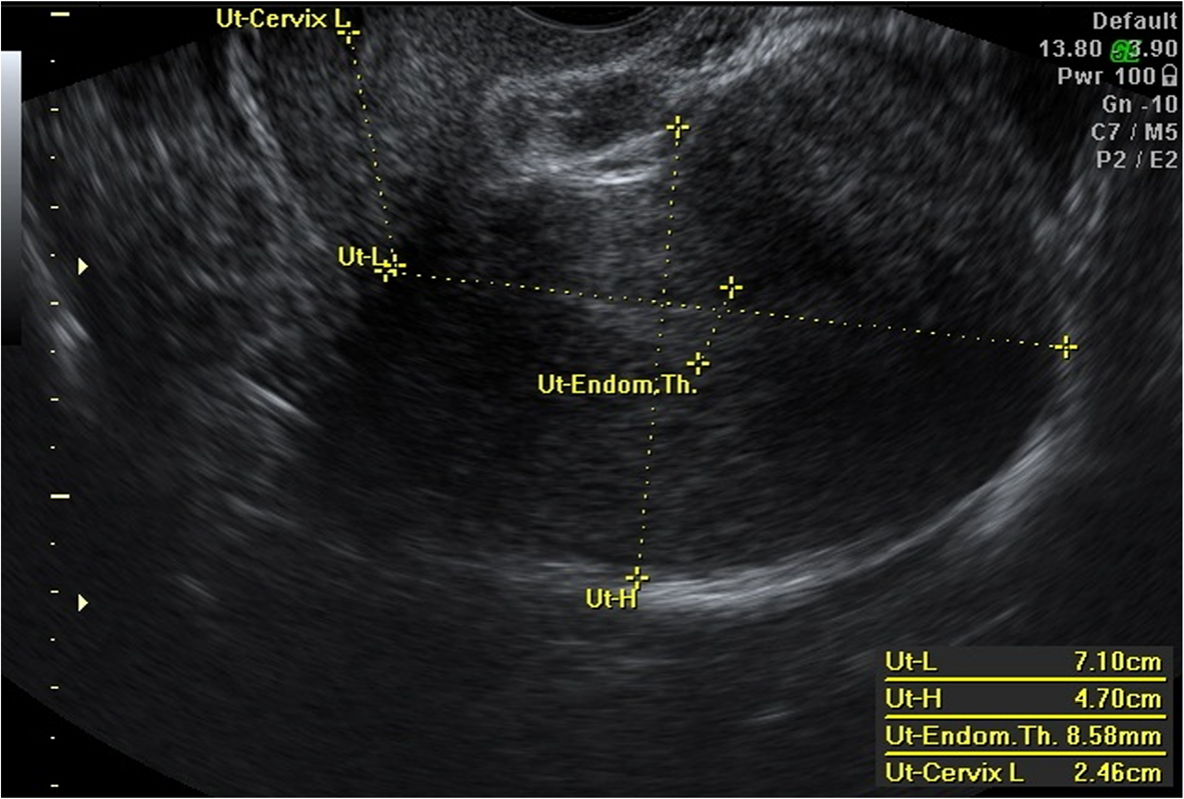
Two-dimensional ultrasound measurement of endometrial thickness

**Fig. 2:**
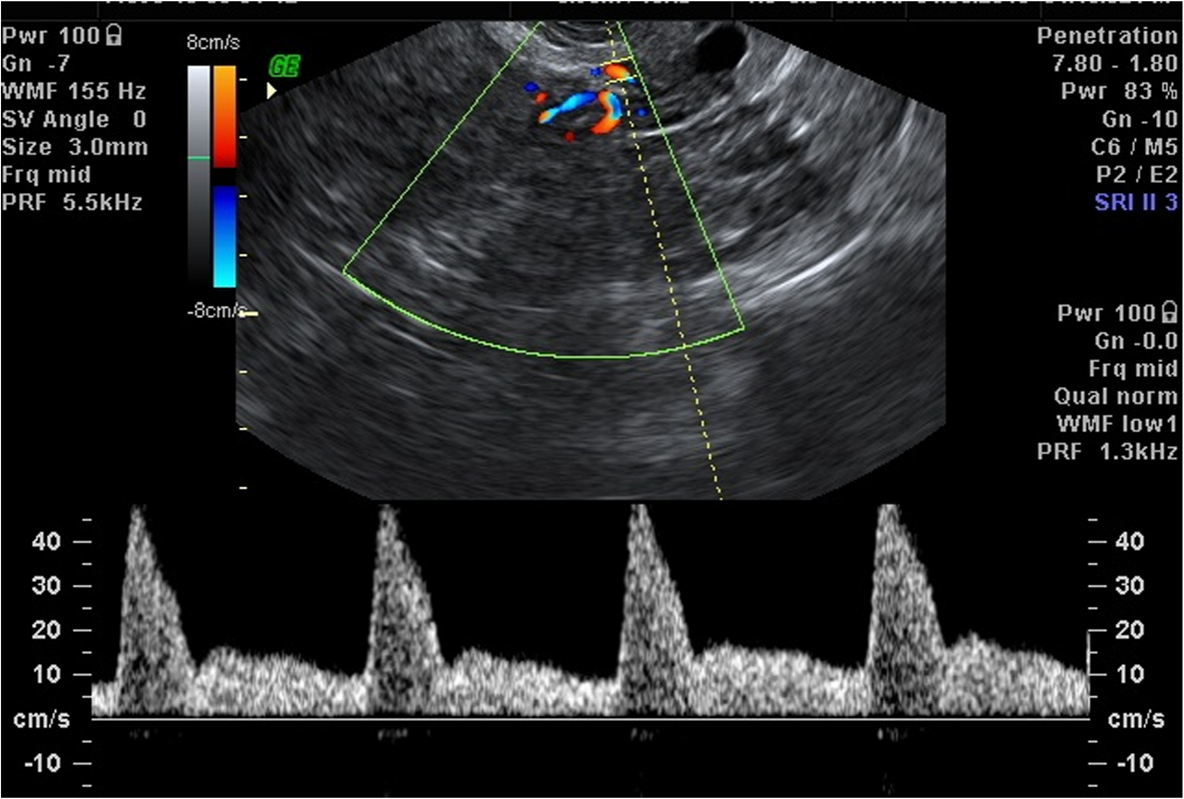
Two-dimensional color Doppler of uterine artery flow velocity waveforms

Transvaginal ultrasound (Voluson 730; Kretz, Zipf, Austria) examinations were performed within 24 h prior to surgery. Using ultrasound in the 2D mode, the endometrial thickness (ET) was measured as the thickest part (double layer) in the sagittal plane (Fig. 1). Then, color Doppler was activated and the flow velocity waveforms were obtained from the ascending main branch of the uterine artery on both sides of the internal os (Fig. 2). Three similar consecutive waveforms of good quality were analyzed, and the averaged right and left uterine artery pulsatility index (PI) and resistance index (RI) were calculated.

**Fig. 3:**
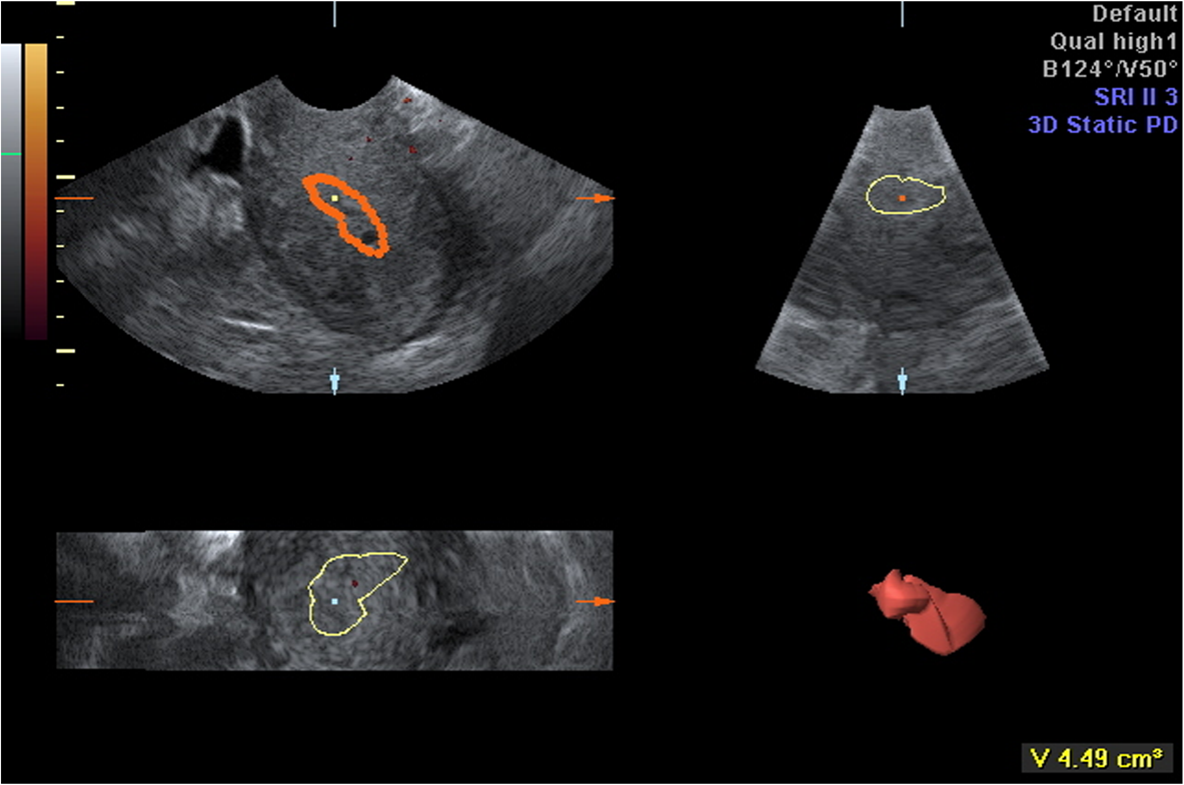
Virtual Organ Computer-Aided Analysis of the endometrium

**Fig. 4:**
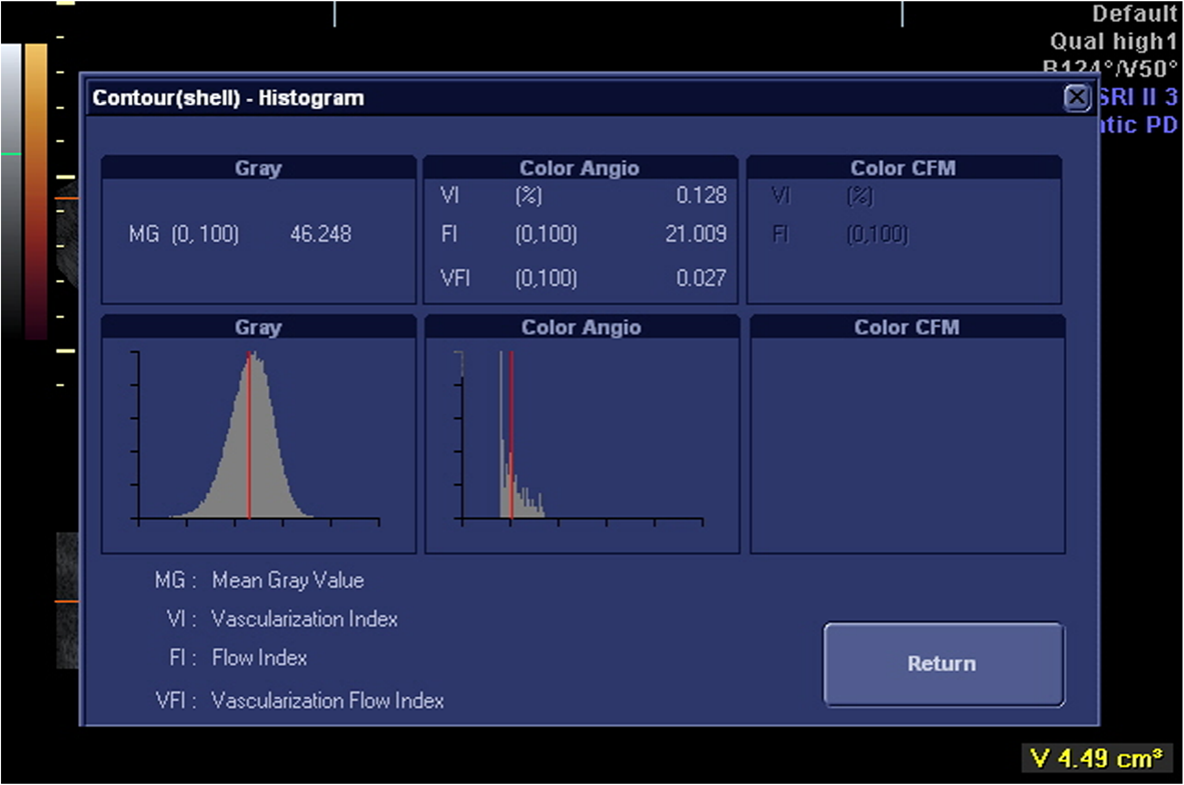
Three-dimensional power Doppler flow indices of the endometrium

The ultrasound was then switched to the 3D mode with power Doppler. The setting conditions for this study were standardized using a frequency at 3–9 MHz, pulse repetition frequency at 0.6 kHz, gain at −4.0, and wall motion filter at low 1. The Virtual Organ Computer-Aided Analysis (VOCAL™) Imaging Program for the 3D power Doppler histogram analysis was used to measure the endometrial volume (EV) and 3D power Doppler indices within the endometrium (Figs. 3 and 4).

Vascularization index (VI) measures the ratio of the number of color voxels to the total number of voxels (%) and represents the presence of blood vessels (vascularity). Flow index (FI) measures the mean power Doppler signal intensity (0–100) and represents the average intensity of blood flow. Vascularization flow index (VFI) is calculated by multiplying VI and FI (0–100) and represents a combination of vascularity and flow intensity.

Hysteroscopic examination was performed routinely before endometrial curettage using a rigid 30° hysteroscope and a 4-mm-diameter diagnostic sheath (Karl Storz GmbH & Co KG, Tuttlingen, Germany). The hysteroscopic diagnosis was based on the following criteria: atrophic endometrium-thin and homogeneous in appearance; endometrial hyperplasia-thickened endometrium, easily indented with pressure, with or without multipolyp appearance; and endometrial carcinoma-irregular growth with or without abnormal vascularization.

Endometrial sampling was carried out by formal dilatation and curettage. The histopathological samples were examined by two senior pathologists who determined the final diagnosis. Ultrasonographic findings were compared with hysteroscopic and histopathologic findings.

### Statistical analysis

Data were expressed as mean ± SD or *n* (%) unless otherwise indicated. Continuous data were compared using Student *t* test or Mann-Whitney *U* test, as appropriate. Receiver-operating characteristic (ROC) curve analysis was used to evaluate the optimal cutoff value of ultrasound markers for prediction of malignant endometrial lesions; based on an equivalent sensitivity and specificity, and the highest value of the area under the curve (AUC). A *P* value <0.05 was considered statistically significant. The Statistical Package for the Social Science (SPSS Inc., Chicago, IL, USA), version 16.0, was used for data analyses.

Sample size calculation reveals that with a margin of error of 4.99 % and a response distribution of 50 %, the confidence level was 52 %; whereas with a margin of error of 9.92 % and a response distribution of 50 %, the confidence level was 84 %.

## Results

Patients’ characteristics and histopathological diagnosis are shown in Table 1. Of the 78 women included in the study, 68 (87 %) had benign endometrium and 10 (13 %) had malignant endometrium (atypical hyperplasia and carcinoma). Hysteroscopic and histopathologic findings were in agreement in almost all cases.

**Table 1:** Patients’ characteristics and histopathological diagnosis (*n* = 78)

Characteristic	Value
Age (y)	47.46 ± 2.94
Parity	4.18 ± 1.18
Weight (kg)	87.79 ± 11.49
Height (cm)	157.10 ± 5.50
BMI (kg/m^2^)	35.53 ± 2.09
Endometrial histopathology; *n* (%)	
Normal endometrium	20 (25.64)
Simple hyperplasia	25 (32.05)
Distorted proliferative	16 (20.51)
Atrophic endometrium	7 (8.97)
Atypical hyperplasia	4 (5.13)
Adenocarcinoma	6 (7.69)

*BMI* body mass index

The age was significantly higher (*P* = 0.032) in patients with malignant endometrium; however, there were no significant differences in the parity (*P* = 0.954), weight (*P* = 0.952), height (*P* = 0.244), or body mass index (*P* = 0.248) between the two groups. The ET (*P* <0.001), EV (*P* <0.001), and endometrial VI (*P* <0.001) and VFI (*P* = 0.043) were significantly increased in patients with malignant endometrium; whereas, the uterine artery PI (*P* = 0.296) and RI (*P* = 0.922) and endometrial FI (*P* = 0.474) were not significantly different between the two groups (Table 2).

**Table 2:** Characteristics of patients with benign and malignant endometrium

Characteristic	Benign (*n* = 68)	Malignant (*n* = 10)	*P* value
Age (y)	46.88 ± 1.95	48.43 ± 2.99	0.032^a^
Parity	4.18 ± 1.21	4.20 ± 1.03	0.954
Weight (kg)	87.76 ± 10.74	88.00 ± 16.44	0.952
Height (cm)	157.38 ± 5.80	155.20 ± 1.81	0.244
BMI (kg/m^2^)	35.29 ± 1.84	36.62 ± 8.42	0.248
ET (mm)	7.615 ± 5.493	21.400 ± 9.489	<0.001^a^
Uterine artery PI	1.847 ± 0.638	2.080 ± 0.755	0.296
Uterine artery RI	1.140 ± 1.583	1.190 ± 0.796	0.922
EV (cm^3^)	3.410 ± 2.728	7.534 ± 3.622	<0.001^a^
Endometrial VI (%)	0.310 ± 0.418	1.005 ± 0.597	<0.001^a^
Endometrial FI (0–100)	22.897 ± 4.547	24.212 ± 9.562	0.474
Endometrial VFI (0–100)	0.100 ± 0.157	0.204 ± 0.072	0.043^a^

*BMI* body mass index, *ET* endometrial thickness, *PI* pulsatility index, *RI* resistance index, *EV* endometrial volume, *FI* flow index, *VI* vascularization index, *VFI* vascularization flow index

^a^Statistically significant

The diagnostic performance of the various ultrasound markers is shown in Table 3. The best marker for discrimination between benign and malignant endometrium was the VI with an AUC of 0.88 at a cutoff value of 0.81 %. The sensitivity, specificity, positive predictive value (PPV), negative predictive value (NPV), likelihood ratio of a positive test (LR+), and likelihood ratio of a negative test (LR−) for endometrial VI at 0.81 % (90 %, 88 %, 53 %, 98 %, 7.50, and 0.11, respectively) were higher than those for ET at 19 mm (80 %, 72 %, 30 %, 96 %, 2.86, and 0.28, respectively), EV at 8 cm^3^ (90 %, 79 %, 39 %, 98 %, 4.29, and 0.13, respectively) and endometrial VFI at 0.22 (60 %, 68 %, 23 %, 92 %, 1.88, and 0.59, respectively).

**Table 3:** Diagnostic performance of ultrasound markers

Parameter	ET	EV	Endometrial VI	Endometrial VFI
Cutoff value	19 mm	8 cm^3^	0.81 %	0.22
AUC	0.73	0.81	0.88	0.67
Sensitivity (%)	80	90	90	60
Specificity (%)	72	79	88	68
PPV (%)	30	39	53	23
NPV (%)	96	98	98	92
LR+	2.86	4.29	7.50	1.88
LR−	0.28	0.13	0.11	0.59

*ET* endometrial thickness, *EV* endometrial volume, *VI* vascularization index, *VFI* vascularization flow index, *AUC* area under receiver-operating characteristics (ROC) curve, *PPV* positive predictive value, *NPV* negative predictive value, *LR+* likelihood ratio of a positive test, *LR* likelihood ratio of a negative test

## Discussion

To the best of our knowledge and review of literature, this is the first study to evaluate the efficacy of 3D ultrasonography and power Doppler for discrimination between benign and malignant endometrium in women with premenopausal bleeding. Several previous studies have evaluated the role of 3D ultrasonography/power Doppler for the investigation of patients with postmenopausal bleeding.

Our results showed that the ET, EV, and endometrial VI and VFI were significantly increased in patients with malignant endometrium than those with benign endometrium; whereas, the uterine artery PI and RI and endometrial FI were not significantly different between the two groups. The best parameter for discrimination between benign and malignant endometrium was the VI with an AUC of 0.88 at a cutoff value of 0.81 %.

In agreement with our results, Mercé et al. [9] and Alcazar and Galvan [10] found that the flow indices were superior to EV for discrimination between endometrial carcinoma and endometrial hyperplasia, and between benign and malignant endometrium, respectively. The best predictor for endometrial cancer was VI. Odeh et al. [11], however, found that EV was superior to the flow indices for discrimination between hyperplasia/malignant endometrium and benign endometrium other than hyperplasia.

Epstein et al. [12] estimated the color content of the endometrium subjectively by choosing the most vascularized area and applying computer analysis to that area. They concluded that power Doppler analysis can contribute to a correct diagnosis of endometrial cancer in women with postmenopausal bleeding. Makled et al. [13] also concluded that 3D power Doppler measurements may be useful for distinguishing between benign endometrial lesions and endometrial carcinoma in women with postmenopausal bleeding.

Kurjak et al. [14] and Kupesic et al. [15] reported the use of volume measurements and power Doppler in diagnosing endometrial and adnexal malignancies; they found significant differences in the volume of malignant and benign lesions. They suggested that a combination of morphologic criteria and 3D power Doppler findings could identify endometrial lesions with sensitivity and specificity of 89 and 97 %, respectively.

Galván et al. [16] found that EV and VI were independently related to myometrial infiltration and tumor stage in endometrial carcinoma; VI was independently associated with tumor grade and EV correlated with lymph node metastases. Saarelainen et al. [17] also suggested that endometrial and, to a lesser degree, myometrial vascular indices and EV correlate with the depth of myometrial invasion in endometrial carcinoma.

Contrary to our results, Lieng et al. [18] did not find differences in 3D power Doppler indices between women with endometrial polyps and endometrial cancer before and after contrast enhanced examination. Opolskiene et al. [19] also concluded that, although 3D power Doppler indices were significantly higher in women with endometrial cancer as compared with those with benign pathology, the diagnostic performance of 3D ultrasound imaging was not superior to that of ET as measured by 2D ultrasound examination.

De Smet et al. [20] analyzed the correlation between EV and myometrial infiltration in a series of 97 women with endometrial cancer. They found that the predicted probability of deep myometrial infiltration increased when the ET increased, while this probability decreased when EV increased. This could be explained by non-linear effects.

The differences in results between our study and previous studies can almost certainly be explained by substantial differences in study populations and study design. There are differences in menopausal status, use of hormone replacement therapy, rate of endometrial cancer, and mix of benign histologies. There are also differences in the methods used to determine diagnostic performance of ultrasound markers. The relatively high rate of endometrial carcinoma and atypical hyperplasia in our study can be explained by exclusion of other causes of premenopausal bleeding in the study population.

## Conclusions

Three-dimensional ultrasonography and power Doppler, especially endometrial VI, may be useful for discrimination between benign and malignant endometrium in premenopausal women with abnormal uterine bleeding before resorting to invasive procedures such as hysteroscopy and endometrial curettage. However, due to our relatively small sample size, further studies in larger series are needed to confirm these data.
